# Prior administration of vitamin K_2_ improves the therapeutic effects of zoledronic acid in ovariectomized rats by antagonizing zoledronic acid-induced inhibition of osteoblasts proliferation and mineralization

**DOI:** 10.1371/journal.pone.0202269

**Published:** 2018-08-20

**Authors:** Bin Zhao, Wenqian Zhao, Yiqiang Wang, Zhao Zhao, Changfeng Zhao, Shue Wang, Chunzheng Gao

**Affiliations:** 1 Department of Orthopedics, The Second Hospital of Shandong University, Shandong University, Jinan, Shandong, People’s Republic of China; 2 Department of Orthopedics, Shouguang Hospital of Traditional Chinese Medicine, Shouguang, Shandong, People’s Republic of China; 3 Department of Traditional Chinese Medicine and Dermatology, People’s Hospital of Shouguang, Shouguang, Shandong, People’s Republic of China; 4 MOH Key Lab of Thrombosis and Hemostasis, Jiangsu Institute of Hematology, The First Affiliated Hospital of Soochow University, Suzhou, People’s Republic of China; 5 Department of Cytology, Qilu Hospital of Shandong University, Shandong University, Jinan, Shandong, People’s Republic of China; 6 Department of Nutrition, Shandong University School of Public Health, Shandong University, Jinan, Shandong, People’s Republic of China; Kyungpook National University School of Medicine, REPUBLIC OF KOREA

## Abstract

Zoledronic acid (ZA) exerts complex influence on bone by suppressing bone resorption, mostly due to the direct osteoclasts inhibition and uncertain influence on osteoblasts. Vitamin K_2_ (VK_2_, Menaquinone-4) as an anabolic agent stimulates bone formation via anti-apoptosis in osteoblasts and mild osteoclasts inhibition. Based on these knowledge, the therapeutic effect of the combined or sequential therapy of VK_2_ and ZA depends on the influence on the osteoblasts, since both cases exert similar inhibitory effect on osteoclasts. In a series of *in vitro* studies, we confirmed the protective effect of VK_2_ in osteoblasts culture, especially when followed by exposure to ZA, and the proliferation and mineralization inhibition induced by ZA towards osteoblasts. For mechanism study, expression of *bcl-2/bax*, *Runx2* and *Sost* in cells were examined. For *in vivo* studies, an osteoporosis animal model was established in rats via ovariectomy (OVX) and subjected to sequential treatment, namely VK_2_ followed by ZA. Bone mineral density (BMD) was measured by Dual energy X-ray absorptionmetry (DEXA), morphology and mechanical parameters by micro-computed tomography (micro-CT), mechanical strength by an electro-hydraulic fatigue-testing machine. The bone calcium, hydroxyproline content, blood lipids were evaluated using microplate technique, and the bone surface turnover was evaluated using the fluorescence in corporation method. It was found that VK_2_ pretreatment partially prevented the inhibition of bone formation caused by ZA, which was reflected by indices like BMD, bone calcium content and bone strength. The underling mechanisms for protection of VK_2_ pretreatment, mainly demonstrated via *in vitro* studies, included inhibiting apoptosis and depressing *Sost* expression in osteoblasts, which in turn improved the osteoporosis therapeutic effects of ZA. These findings suggested that pretreatment with VK_2_ before ZA therapy might serve a new long-term therapy protocol for osteoporosis.

## Introduction

Osteoporosis is caused by imbalance between new bone formation and bone absorption that are dominated by osteoblasts and osteoclasts respectively. In specific, outpacing of osteoclastic bone resorption over osteoblastic bone formation results in a highly porous structure in the bone. All treatments for this disease aim at restoration of the original innate balance by enhancing bone formation or/and inhibiting bone absorption. Undoubtedly, osteoblasts and osteoclasts are popular targets for such a purpose. However, Frost reported that mere depressing osteoclasts activity or enhancing osteoblast activity was not enough to cure the osteopenia that accompanied osteoporosis [1], since, though the anticatabolic or anabolic agents can improve bone mass, the mass tended to decrease again when the treatment was withdrawn, or even when the treatment was still continuing [2, 3]. Thus osteoporosis as a clinical problem could not be resolved by a quick-fix. Rather, a long term strategy with minimized side effect should be anticipated. A seemingly obvious way to prevent some of the side-effects anticatabolic agents would be to combine treatment with anabolic and anticatabolic agents, either as simultaneous or sequential administration.

Bisphosphonates (BPs) are a major class of anti-bone resorption drugs that have been widely used for treating osteoclast-mediated bone loss such as postmenopausal osteoporosis, complications of cancer metastasis, or osteolytic bone diseases like Paget`s disease. By linking to hydroxyapatite, BPs inhibit osteoclast recruitment and bone turnover [4]. BPs exert their functions mainly by impairing osteoclast function, which is reflected by morphologic changes of osteoclast cytoskeleton, disruption of ruffle borders, and apoptosis [5]. Zoledronic acid (ZA) is a representative of new generation nitrogen-containing BPs, and possesses higher mineral-binding affinity and stronger anti-resorption activity than other BPs. ZA effectively suppresses osteoclast-mediated bone resorption by inhibiting the farnesyl pyrophosphate synthase action in the mevalonate pathway [6]. In addition, the feasibility of dosing as 5 mg infusion once a year ensures a favorable patient adherence to ZA. These advantages endow ZA with promises for restoring bone mineral density (BMD) and bone strength in osteoporosis, or reducing fragility fracture incidence in postmenopausal women.

However, with the spreading use of BPs, including ZA, evidence of side effects of BPs are also accumulating. While majority researches confirmed the effective and safety of a single dosage of ZA for osteoporosis treatment (e.g. a single injection of 4mg or 5mg ZA has been reported to inhibit bone turnover effectively for 12 months [7] or 18 months [8]), a couple of studies brought up the negative effect of ZA treatment. In a rat bone defect model, ZA given at doses equivalent to human osteoporosis treatments showed no effect on properties of newly formed bone and, on the contrary, high dose ZA disrupted collagen and apatite crystal organization via decreasing the hydroxyproline-to-proline ratio, thereby hindered bone healing [9]. When applied locally at grafted bones, ZA directly blocked bone metabolism and osteointegration, hence decreased the new bone formation [9, 10]. Another study showed that the risk of atypical fracture increased along with the yearly ZA administration [11]. The negative effects of ZA were readily revealed in in vitro system. ZA of 100μmol/L added to osteoblast precursor cell line MC3T3 cells induced apoptosis directly [12], while ZA of 1μmol/L and 5μmol/L (equivalent to human osteoporosis treatment) caused cytotoxic effects towards the osteosarcoma MG63 cells manifested as reduced cell viability, total protein production, alkaline phosphatase gene expression, or osteocalcin activity [13]. Such negative effect of ZA toward osteoblasts proliferation and functions was also detectable at much lower concentrations (e.g. above 10^−8^ mol/L) [14]. Based on such updates, providing an effective and safe protocol for long-term ZA treatment for osteoporosis remains a challenge. On the other hand, vitamin K_2_ (VK_2_, either Menaquinone-4 or Menaquinone-7 in this context) exerts beneficial or anabolic effects towards osteoblasts, as reviewed in a couple of recent articles [15, 16]. In biochemical context, VK_2_ is a cofactor of γ-carboxylase that converts three glutamic acid (Glu) residues in osteocalcin (OC) to γ-carboxyglutamic acid (Gla), so γ-carboxylation of OC is likely one of the therapeutic mechanisms for VK_2_ [17, 18]. Otherwise, when undercarboxylated OC is used for bone structural constitution, its ability to bind the mineral hydroxyapatite is comprised. Thus, insufficiency of VK_2_ in nutrition will increase the concentration of undercarboxylated OC in serum and increase the risk of fractures [18]. Another mechanism for VK_2_ to treat osteoporosis is that VK_2_ inhibits apoptotic cell death of osteoblasts [19]. In detail, VK_2_ reduces the expression of proapoptotic agents *Fas* and *Bax* in a dose-dependent manner in osteoblasts. The increases of growth differentiation factor-15 (GDF-15) and stanniocalcin 2 [20] are also connected with VK_2_-induced activation of promoters that are involved in differentiation and proliferation of osteoblasts. Lastly, VK_2_ suppressed the osteoclastogenesis by suppressing NF-κB activation [21]. The beneficial effect of VK_2_ was also noticed in animal models [22]. Based on such evidence, though in presence of controversial ones, VK_2_ intake had been proposed to be helpful for osteopenia or osteoporosis victims [23, 24]. Also promoted by above observations and clues, we proposed that VK_2_ and ZA were promising candidates for a concomitant and long-term therapy for osteoporosis. More specifically, we proposed that prior administration of VK_2_ might boost the osteoblasts functions or equip the osteoblasts with an ability to antagonize the inhibiting effect of ZA on osteoblast proliferation or mineralization. To test this hypothesis, in vitro cell culture and an animal osteoporosis model were utilized to compare the effect of different protocols concerning VK_2_ and/or ZA administration.

## Materials and methods

### Ethic statement

Animal experimental protocols observed the Guidelines on the Humane Treatment of Laboratory Animals (Ministry Of Science and Technology of China, 2006) and was approved by the Animal Experimental Ethics Inspection of Preventive Medicine Research Project of Shandong University (Permit Number: 20150902). Each effort was made to minimize uneasiness of animals during all processes.

### Isolation and primary culture of murine osteoblasts

Three-day-old neonatal mice of Kunming strain were obtained from Experimental Animal Center of Shandong University. Osteoblasts were obtained from mouse calvaria using the method of collagenase-pancreatic enzyme digestion as detailed in reference [25]. Isolated cells were cultured in Dulbecco’s Modified Essential Medium (DMEM) supplemented with 10% FBS, 100U/mL penicillin and 100μg/mL streptomycin and incubated at 37°C in 5% CO_2_/95% air. The culture medium was replaced every other day. Confluent cells were dispersed with 0.25% trypsin plus 0.05% EDTA in buffered saline (pH 8.0) and then transferred to new culture flasks in a split ratio of 1:2. After two passages, the cells were identified by the characteristic features to be desired osteoblast cells, and the alkaline phosphatase staining method was utilized in this procedure as well (Figure A in S1 File). Alizarin Red-S staining was further utilized to identify osteoblast and its purity in culture.

### Treatment of cells with VK_2_ and/or ZA and cellular/molecular assay

**Fig 1:**
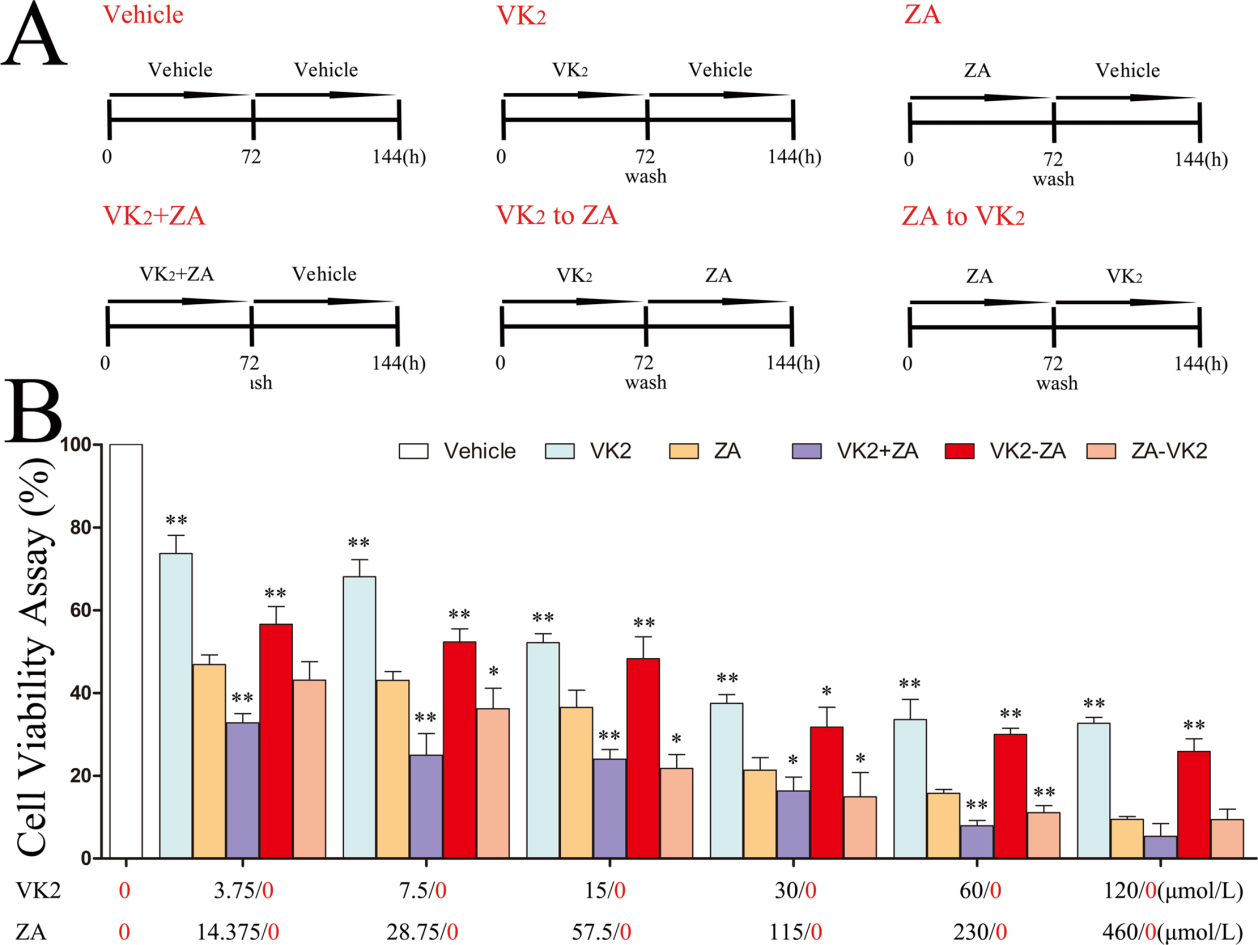
Effect of treatment with VK_2_ and/or ZA on osteoblast viability in vitro. (A) Illustration of the protocols of the drug administration. (B) Effect on cell viability by VK_2_ and/or ZA at different concentrations, alone or in combination. Dunnett’s two-tailed t-test was used for multiple comparisons of ‘ZA alone’ group against each other group. *p<0.05, **p<0.01.The concentrations of each drug was given below the columns as N/0, indicating that that specific drug was present at N μmol/L or absent as requested by each specific protocol(Fig 1B).

After two passages, osteoblast cells were seeded at 2×10^3^ cells/well in 100μL complete growth medium in 96-well plate and allowed to attach. Cells were exposed to different concentrations of VK_2_ (Menatetrenone-4. Shanghai Reson Biotech Co., Ltd, Shanghai, China) or ZA (Huifengda Chemical Co., Ltd, Jinan, China), which were detailed in Results and figure legends. Untreated wells were set as control cells. After 72 hours of incubation, the MTT method was used to assess the number or viability of cells in culture. In brief, the cultures were washed twice with PBS (pH 7.4) and replenished with DMEM containing MTT and incubated for another 4 hours. After removing the medium carefully, 100μL DMSO was added to each well, and a microplate reader (Multiskan MK3, Thermo Fisher Scientific, Pittsburg, MA) was used to measure absorbance at 570nm (A570). Cell viability percentage was calculated as (A570 of treatment—A570 of blank)/(A570 of control—A570 of blank)x100%. With all viability percentages under different treatment, 50% inhibition (IC50) was calculated by GraphPad Prism 5.0 for Windows (GraphPad Software, Inc,. La Jolla, CA). Then, the effect of combinational or sequential administration of 2-fold serial concentrations of VK_2_ and ZA was studied in a six-day timeline (Fig 1A). Based on predetermined IC50, VK_2_ concentrations were set at 3.75, 7.5, 15, 30μmol/L (i.e. IC50), 60,120μmol/L, and ZA at 14.375, 28.75, 57.5, 115μmol/L (i.e. IC50), 230μmol/L, 460 μmol/L. Again, control wells were set as untreated cells. At the end of 144-hour incubation, cell viability was determined as above, and a combination index (CI) was calculated by CalcuSyn program version 2.1 (Biosoft, Cambridge, UK) based on the analytical method of Chou and Talalay [26]. In such situation, the CI index at the 50%, 75% and 90% effective dose (ED) were calculated, a CI<1 indicated synergy effect, CI = 1 additive effect, and CI>1 antagonistic effect. The experiments were repeated three times.

### Alizarin Red-S staining assay

To observe the morphological feature of osteoblasts, Alizarin Red-S staining method was used. Briefly, 2×10^4^ cells/well were seeded in complete growth medium in 24-well plate and cultured for a total of 144 hours under treatment with different protocols (Fig 1A). At the end of culture, cells were rinsed with PBS and fixed with 70% ethanol for 10 min at 4°C, followed by staining with Alizarin Red-S (1%, PH 4.2,Tris-HCL) for 30 min at room temperature. Excess stain was carefully removed with distilled water. An inverted phase contrast microscope (Eclipse TS100, Nikon, Tokyo, Japan) was used to observe the morphology of and calcium deposition in cells. The images were captured and analyzed with NIS-Elements F (Nikon, Tokyo, Japan).

### Calcium matrix deposition assay

Arsenazo III calcium assay was used to evaluate the quantity of calcium matrix deposition as previously described [27]. In brief, cells were seeded in 24-well plate at 2×10^4^ cells/well and cultured for a total of 144 hours (Fig 1A) before harvested into PBS and sonicated with a SONOPULS mini20 (Bandelin, electronic GmbH & Co.KG, Berlin. Germany). Arsenazo III (Maclin, Shanghai Maclin biochemical Co.,Ltd, Shanghai, China) was added into the lysate, and the absorbance was measured at 650nm wavelength with a microplate reader [28].

### Reverse transcription and real time-PCR assay

4×10^4^ cells/well were seeded in complete growth medium in 6-well plate and cultured for a total of 144 hours under treatment with different protocols (Fig 1A). VK_2_ concentration was set at 7.5μmol/L and ZA at 28.75 μmol/L. Gene expression assays in cultures that were treated at other concentrations of VK_2_ and ZA were not attempted. At the end of culture as above, cells were harvested and total RNA was isolated using RNAiso Plus (Takara, Otsu, Japan) according to the manufacturer`s instruction and further purified with the gDNA Eraser (Takara). After quantitation, total RNA was reverse transcribed using the PrimeScript RT Enzyme Mix I (Takara) at 37°C for 15min. The first stranded cDNA was subjected to PCR for different genes using the SYBR green method in a Mastercycler realplex2 machine (Eppendorf, Hamburg, Germany) with cycling condition as follows: 95°C, 30 s, subsequently 40 cycles of 95°C, 5 s, 60°C, 34 s. The following primers were purchased from GenScript Co., Ltd. (Nanjing, China). *GAPDH*, AACTTTGGCATTGTGGAAGG and GGATGCAGGGATGATGTTCT; *Bcl-2*, CTCGTCGCTACCGTCGTCGTGACTTCG and CAGATGCCGGTTCAGGTACTCAGTC; *Bax*, ACCAGCTCTGAACAGATCATG and TGGTCTTGGATCCAGACAAG; *Runx2*, GACTGTGGTTACCGTCATGGC and ACTTGGTTTTTCATAACAGCGGA; *Sost*, ATCTGCCTACTTGTGCACGC and TCATAGGGATGGTGGGGAGG. After amplification, Ct was obtained for each reaction. GAPDH was used as reference gene and the relative gene expression levels of each target gene in a specific sample was calculated as 2^-ΔCt^ were ΔCt = Ct_gene_-Ct_GAPDH._ Then the expression of the gene in experimental group was calculated by 2^-ΔΔCt^ method, where ΔΔCt = ΔCt_exp_-ΔCt_control_.

### Treatment protocols in animal model

An ovariectomy (OVX) model was utilized to study the effect of VK_2_ and/or ZA on bone biology in vivo. In brief, seventy-six 20-week-old healthy female SPF Wistar rats (weighting 250–330 g) were purchased from the Animal Center of Shandong University. All rats were housed in individual cages under controlled temperature (around 22°C) and relative humidity (40%–60%), with a 12-h light-dark cycle, and were allowed free access to distilled water and standard chow supplemented with 11.7 g calcium plus 3 mg vitamin K per kilogram. The rats were divided into seven groups according to a random number table method: (A) sham-operated (n = 10), (B) OVX (n = 11), (C) VK_2_ (n = 11), (D) ZA (n = 11), (E) VK_2_+ZA (n = 11), (F) VK_2_ to ZA (n = 11), and (G) ZA to VK_2_ (n = 11). Ovariectomy or sham operations were performed under anesthesia via an intraperitoneal dosing of pentobarbital sodium (9mg/animal, Solarbio, Beijing, China) after grouping. Two weeks after OVX, the animals were subjected to treatment as grouped and detailed below. During the first 6-weeks term of treatment, the VK_2_, VK_2_+ZA and VK_2_ to ZA groups received VK_2_ every day, while the ZA, VK_2_+ZA and ZA to VK_2_ groups received a single dose of ZA at the beginning of this term. After entering the second 6-weeks term, the VK_2_, VK_2_+ZA and ZA toVK_2_ groups received VK_2_ every day, while the ZA, VK_2_+ZA and VK_2_ to ZA groups received a single dose of ZA at the beginning of this term. VK_2_ (MK-4, menatetrenone soft capsules, GLAKAY, Eisal, Tokyo, Japan) was prepared with corn oil to 30 mg/mL and administered intragastrically at 30 mg/kg/d with a syringe gauge. ZA (Novartis Pharma Schweiz AG, Stein, Switzerland) was administered as a single dose via intraperitoneal injection at 0.1 mg/kg. Doses of VK_2_ and ZA were based on previous studies [29, 30]. For surface-based bone turnover assay, rats were injected (i.p.) with tetracycline (Ruitaibio, Beijing, China. 30mg/kg) on the 15th and 14th day before sacrifice, and injected (i.p.) with calcein (Solarbio. 10mg/kg) on the 4th and 3rd day before sacrifice. At the end of totally 12 weeks of treatment, sixty rats completed the experiment, and animals were anesthetized and euthanized by carotid artery bleeding. The other 16 rats dropped out the experiment when they lost more than 40 percent of their weight in one week and euthanized by carotid artery bleeding.

### Blood lipids analysis

Blood lipids in harvested serum of rats were measured using commercial kits specific for triglyceride, total cholesterol, high density lipoprotein cholesterol and low density lipoprotein cholesterol, respectively, all from Nanjing JianCheng Bioengineering Institute.

### BMD measurement

The femurs collected from rats were packed separately in saline-soaked gauze to maintain moisture at room temperature and scanned with DEXA bone densitometer machine (Norland XR-600, Norland Cooper Surgical, Trumbull, CT). The femur’s position was adjusted to ensure that the major axis of the femoral condyles was perpendicular to the testing platform. BMD and bone mineral content (BMC) were calculated using the accompanied small animal imaging software.

### Micro-CT scanning

To evaluate morphological status in femurs, a Siemens preclinical Inveon PET/SPECT/CT (Siemens Medical Solutions USA, Knoxville, TN, USA) was utilized. The spatial resolution was set to 8.5 μm with a voxel size of 17.2× 17.2 × 17.2 (μm), and the tube voltage and current is 80 kV and 500 μA, respectively. The resolution was set to medium (1000 projections each), increment were set to 17.2 μm, and the region of interest area was 0.225 mm proximal to the growth plate, that is,100–250 layer proximal to the butterfly area. Three-dimensional imaging was performed using COBRA Exxim (licensed to Siemens). Morphological analysis was carried out using a Inecon Research Workplace, and various trabecular bone parameters were recorded, including bone specific surface (BS/BV; 1/mm), bone volume fraction (BV/TV; %), trabecular thickness (Tb.Th; μm), trabecular number (Tb.N; 1/mm), trabecular separation (Tb.Sp; μm) and trabecular bone pattern factor (Tb.Pf; 1/mm). The cross-sectional moment of inertia (CSMI) at the lower-middle third of the femur was also measured.

### Three-point blending test

After micro-CT scanning, the femurs were wrapped with saline-saturated gauze and a three-point bending test was performed using a compact servohydraulic fatigue testing system (INSTRON 8801,Instron industrial products, Grove city, PA, USA) at room temperature and 55% humidity. Briefly, each femur was placed on the two supports of the test apparatus that were 20 mm apart (L). The load was applied to the lower-middle third of the femur in a postero-anterior direction, and the loading speed was 2 mm/min. The time, deflection (d), ultimate load (UL) as well as yielding point (elastic load, EL) were recorded. Bone stress and Young’s modulus (E) were calculated using specific equations: ultimate stress (US) = (UL×L×H)/(8×CSMI), where H is the outside diameter parallel to the loading direction, and L is the support span (20 mm); elastic stress (ES) = (EL×L×H)/(8×CSMI); Young’s modulus, E = (EL×L^3^)/(48×CSMI×d).

### Bone calcium and hydroxyproline contents assay

After the three-point bending test the broken femurs were dehydrated. The proximal femurs were then carbonized by burning to ash at 550°C for 5 hours. The ashes were dissolved in 6 mmol/L HCl, and the content of calcium was measured using a Calcium Assay Kit (Nanjing JianCheng) following the procedure provided by the manufacturer. Meanwhile, the distal femurs were carefully scraped to remove periosteal and bone marrow tissue, then hydrolyzed in 6 mmol/L HCl at 100°C for 7 hours. Then the pH of hydrolysates was adjusted to 6.0–6.8 and the hydroxyproline content was measured using a Hydroxyproline Kit (Nanjing JianCheng).

### Bone histomorphometry index (surface-based bone turnover) assay

The tibias collected from the rats were dehydrated by alcohol, and then were prepared for embedding in a light cure acrylic resin (NMS-SL, Nanjing Mucyte BioTech Co,.Ltd, Nanjing, China). The central sagittal histological sections were obtained using a LEICA RM2145 (Leica Biosystems Nussloch GmbH, Nussloch, Germany). The fluorescence of tetracycline and calcein was observed under a fluorescence microscope (Eclipse 90i, Nikon, Tokyo, Japan), and the percent labeled perimeter (%L.Pm, %) were measured with the Digimizer Image Analysis Software (Version 4.2.6.0, MedCalc Software bvba), and mineral apposition rate (MAR, μm/d) and bone formation rate (BFR, μm/d*%) were calculated accordingly.

### Statistical analysis

When applicable, all data were presented as means ± standard deviation. Comparison of data between groups was performed using a one-way analysis of variance (ANOVA), and Dunnett’s two-tailed t-test was used when making multiple comparisons to the ZA (in vitro) or OVX group (in vivo). The Student-Newman-Keuls (SNK) method was then used for multiple comparisons among the protocols (in vitro) or treatment groups (in vivo) that were found to be statistically significant in previous tests. Rank the data then using a one-way analysis of variance again, or nonparametric test was performed directly when the variances were unequal. All statistical analyses were performed using the Statistic Package for Social Science (SPSS 19.0). Probability values <0.05 were considered to be statistically significant.

## Results

### Differential responses of cultured osteoblasts to various VK_2_/ZA treatment protocols

**Fig 2:**
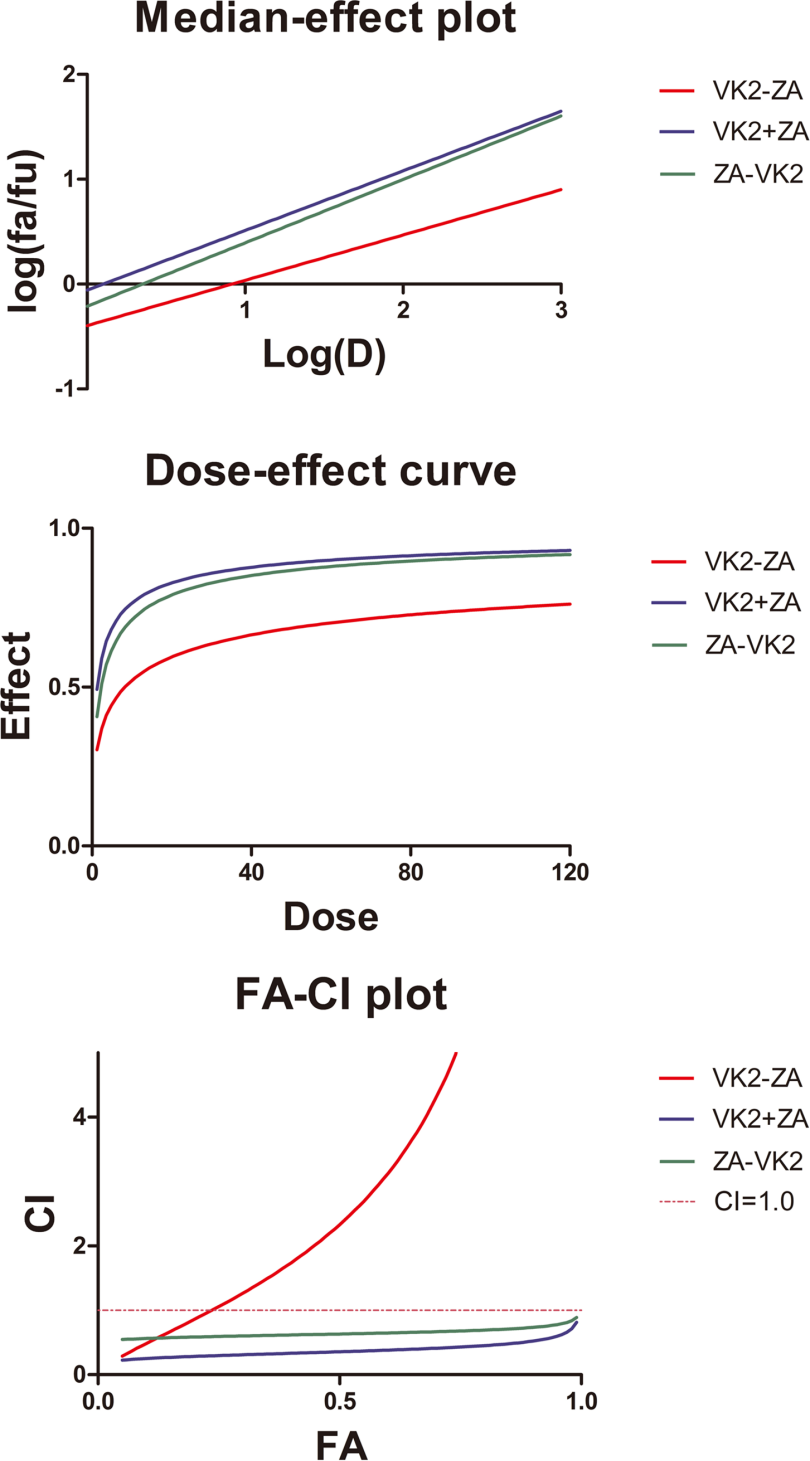
The combination indexes. Plot showing calculation for combination indexes (CI).

**Fig 3:**
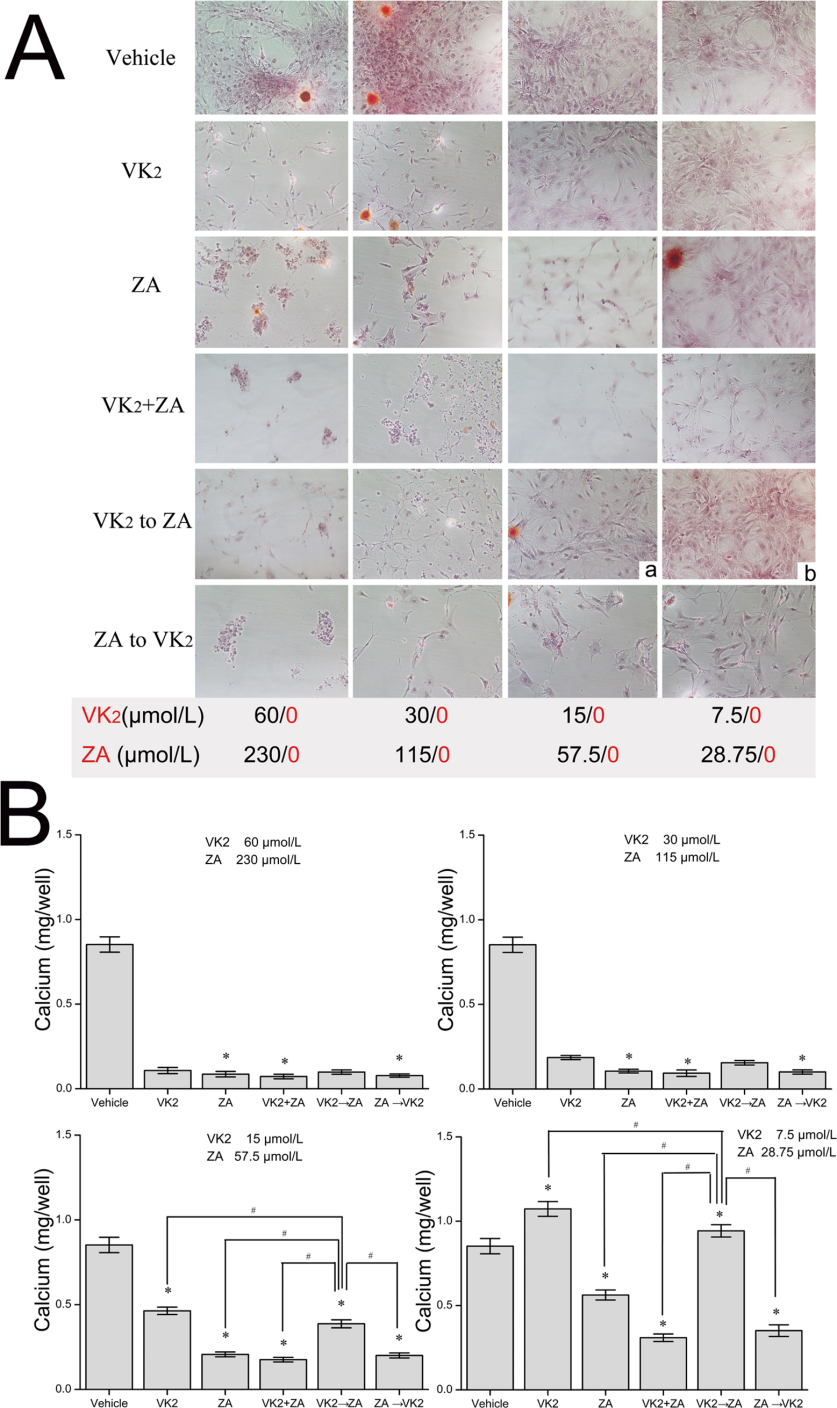
Morphology of osteoblasts and mineralization outcomes after treatment of various protocols. Alizarin Red-S staining method was used after 144 hours of culture. The concentrations of each drug was given below the columns as N/0, indicating that that specific drug was present at N μmol/L or absent as requested by each specific protocol. Dunnett’s two-tailed t-test was used in mineralization outcomes when making multiple comparisons to the vehicle group (Nonparametric test was performed directly when the variances were unequal), *p<0.05 vs vehicle. The SNK method was then used for multiple comparisons among the treatment groups that were found to be statistically significant in previous tests. ^#^p<0.05 vs VK_2_ to ZA.

When osteoblasts were subjected to treatments with either VK_2_ or ZA alone (Fig 1A), cell viability manifested a dose-dependent decrease in both cases at 72 hours, while the cell viability was much lower in ZA group than in VK_2_ group at comparable concentrations (in term of IC50). This suppression was more significant when both agents were present at the same time (VK_2_+ZA, Fig 1B). However, prior administration with VK_2_ before ZA manifested a better cell viability than those treated with ZA alone, while treatment with VK_2_ after ZA administration did not incur such protection (Fig 1B). When a combination index (CI) was calculated to check if synergy or antagonistic effect existed for combinational or sequential administration, it was found that that prior administration with VK_2_ followed by ZA generated antagonistic effect (CI = 2.33063 at the ED50; CI = 5.17332 at the ED75; CI = 11.56291 at the ED90), while the combined administration (CI = 0.35692 at the ED50; CI = 0.43031 at the ED75; CI = 0.52238 at the ED90) or prior administration with ZA followed by VK_2_ generated synergy effect (CI = 0.63323 at the ED50; CI = 0.68027 at the ED75; CI = 0.73588 at the ED90) (Fig 2). That is to say, prior administration of VK_2_ antagonize the effects of following ZA on osteoblasts, but ZA to VK_2_ and ZA+VK_2_ generated synergy effect thus were even more harmful than ZA alone. In accordance with cell viability data revealed with above MTT assay, microscopic observation assisted with Alizarin Red-S staining confirmed the damage pattern of VK_2_/ZA treatments on osteoblasts. Again, ZA and VK_2_+ZA treated cells manifested severe damage or proliferation inhibition, while VK_2_ to ZA protocol least (Fig 3A). Besides that, *ZA* above 28.75μmol/L (1/4 IC50) inhibited the osteoblast mineralization as characterized by the lower calcium matrix deposition (Fig 3B). On the contrary, VK_2_ at the comparable concentrations (1/4 IC50, i.e. 7.5μmol/L) enhanced the mineralization significantly. The VK_2_ to ZA protocol also enhanced mineralization. Based on the above evidence, prior administered VK_2_ can maintain osteoblasts proliferation during the following ZA exposure, and contributed to enhanced mineralization in a lower concentration range.

### Differential effect of VK_2_/ZA treatment protocols on gene expressions in osteoblasts

**Fig 4:**
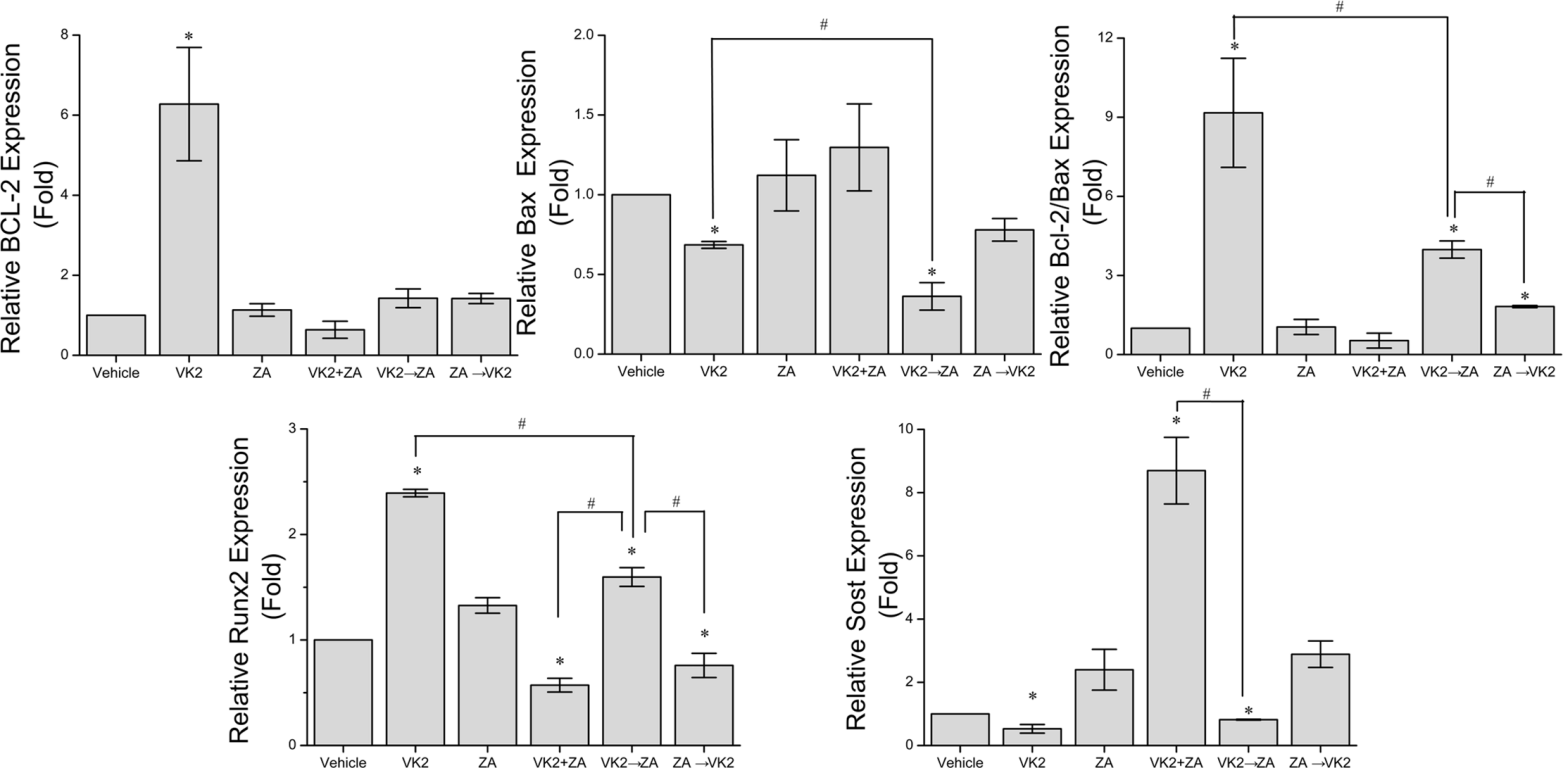
Expression of *Bcl-2*, *Bax*, *Runx2* and *Sost* gene at mRNA levels in cells treated with different protocols. Dunnett’s two-tailed t-test was used when making multiple comparisons to the ZA group, *p<0.05 vs ZA. The SNK method was then used for multiple comparisons among the treatment groups that were found to be statistically significant in previous tests. ^#^p<0.05 vs VK_2_ to ZA.

To explore the possible molecular mechanism(s) for VK_2_ and ZA interactions, four genes involved in osteoblast functions were measured for their expression in osteoblasts cells after treatment as above. VK_2_ alone induced a significant increase of Bcl-2/Bax ratio compared with control cells, while VK_2_+ZA did not (Fig 4). Actually VK_2_+ZA decreased Bcl-2/Bax ratio (below 1.0). However, when VK_2_ and ZA were administered sequentially (VK_2_ to ZA), the rescue effect of VK_2_ was much better. On the contrary, when administered after ZA, VK_2_ failed to rehabilitate the osteoblasts. With the other two genes, *Runx2* and *Sost*, which are supposed to be restricted to osteoblasts, VK_2_ slightly up-regulate the expression of *Runx2* (above 1.0) and down-regulated the exression of *Sost* (below 1.0) than in control cells, while ZA significantly increased expression of *Sost* (above 1.0) and slightly increased thr expression of *Runx2* (above 1.0). In all three combinations of VK_2_ and ZA, VK_2_ to za protocol manifested the tendency of up-regulating *Runx2* expression and down-regulating *Sost* expression in comparison with ZA alone, while the VK_2_+ZA or ZA to VK_2_ protocol failed to do so.

### Enhanced protective effect of prior VK_2_ dosing in OVX models

Finally, the potential benefit of sequential dosing of VK_2_ prior to ZA treatment was examined using the OVX animal models. VK_2_ or ZA alleviated the bone loss in OVX animals, as reflected by the calcium contents in femurs (Table 1). When administered prior to ZA treatment, VK_2_ enhanced the effect of ZA, while simultaneous or post-ZA VK_2_ administration did not. None of other biochemical indices, including the body weights, triglyceride, total cholesterol, high or low density lipoprotein cholesterol and hydroxyproline contents, manifested significant variations among these groups (Table 1), indicating that the bone calcium content was the main target affected by relevant protocols in OVX models. Corresponding to that notion, OVX animals showed a defeat feature in BMD and BMC values in comparison with sham group. Sequential protocol (VK_2_ to ZA) improved the BMD values over VK_2_, ZA and ZA to VK_2_ protocols, while simultaneous protocol failed to improve the BMD and BMC values over OVX models. The BMD and BMC as detailed in Table 2.

**Table 1:** Body weight, surface-based bone turnover data, bone calcium content, bone hydroxyproline content and physical features of bones.

	Sham (n = 8)	OVX (n = 7)	VK_2_ (n = 8)	ZA (n = 11)	VK_2_+ZA (n = 9)	VK_2_ to ZA (n = 9)	ZA to VK_2_ (n = 8)
Body weight (g)							
Original	300.00±35.46	298.00±59.28	313.75±38.52	284.55±44.13	272.22±50.69	275.56±41.87	278.75±50.27
Final	346.25±21.34	380.00±41.63	352.50±18.32	343.64±35.01	345.56±48.76	344.44±35.75	336.25±40.69
Surface-based bone turnover data							
%L.Pm (%)	2.09±0.29*	4.60±0.32	6.00±0.75*_def_	3.99±0.56*_cef_	2.10±0.27*_cdf_	5.27±0.35*_cde_	4.65±0.64
MAR (μm/d)	0.18±0.03*	0.38±0.03	0.49±0.05*_ef_	0.33±0.04	0.18±0.04*_cf_	0.43±0.03*_ce_	0.38±0.04
BFR (μm/d*%)	0.38±0.10*	1.74±0.26	2.93±0.56*_def_	1.35±0.34*_cef_	0.38±0.12*_cdf_	2.26±0.32*_cde_	1.97±0.12
Bone calcium and hydroxyproline content							
Bone calcium content (mg/g)	275.61±5.50*	249.63±4.60	261.21±6.46*_f_	261.78±9.13*_f_	256.82±8.71	271.86±5.54*_cd_	259.58±4.66
Bone hydroxyproline content (mg/g)	6.33±0.49	5.74±1.10	6.00±0.50	5.91±0.85	5.51±0.48	5.90±0.85	5.69±0.83
The trabecular bone parameters of the distal metaphysis							
BV/TV (%)	45.66±5.17*	19.16±3.91	28.63±4.06*_f_	30.06±5.89*_f_	21.24±3.33	40.22±5.96*_cdg_	27.27±6.34*_f_
BS/BV (1/mm)	18.84±5.78*	27.01±5.02	23.06±3.34	22.21±6.82	25.41±3.57	20.18±4.74*	23.93±3.70
Tb.Th (μm)	104.94±12.77	87.90±15.82	94.37±14.94	93.81±14.38	97.19±13.90	100.46±9.79	84.05±11.71
Tb.N (1/mm)	4.37±0.46*	2.19±0.35	3.06±0.38*_f_	3.22±0.53*_f_	2.44±0.20	4.01±0.53*_cdg_	3.24±0.53*_f_
Tb.Sp (μm)	125.75±19.33*	377.98±71.36	237.50±44.07*_f_	224.50±51.10*_f_	324.36±33.37	152.58±32.99*_cdg_	231.27±49.22*_f_
Tb.Pf (1/mm)	-4.24±1.17*	3.05±0.27	1.38±0.66*_f_	1.26±0.68*_f_	1.79±0.41*	-1.19±0.84*_cdg_	1.23±0.79*_f_
The bone biomechanical properties							
Elastic load (N)	123.86±16.71*	87.86±9.93	100.33±7.28	98.14±9.48	94.80±8.76	109.67±18.12*	92.41±15.55
Elastic stress (N/mm^2^)	168.32±18.88*	123.78±12.86	145.82±13.48*	141.16±15.47	133.26±13.10	152.28±13.28*	131.91±20.00
Young`s modulus (N/mm^2^)	9457.03±1028.36*	7781.72±785.06	8741.48±601.04	8749.52±881.30	8434.97±541.15	9181.14±948.32*	8358.34±787.32
Ultimate load (N)	132.51±12.75	122.87±7.29	128.98±10.67	129.06±5.50	128.14±7.49	131.55±13.97	129.07±17.52
Ultimate stress (N/mm^2^)	209.65±15.00*	173.25±4.83	189.52±15.34	180.20±11.21	174.10±16.14	198.76±8.99*	173.27±13.62
CSMI (mm^4^)	5.46±0.64	5.75±0.68	5.41±0.52	5.51±0.38	5.53±0.37	5.36±0.56	5.41±0.70

Data are presented as the mean±SD. P<0.05 is considered statistically significant. A comparison of data between groups was performed using a one-way analysis of variance (ANOVA). Rank the data then using a one-way analysis of variance again, or nonparametric test was performed directly, when the variances were unequal. Dunnett’s two-tailed t-test is used when making multiple comparisons to the OVX group

* p<0.05 vs OVX. The SNK method is then used for multiple comparisons among the treatment groups that are found to be statistically significant in previous tests. _c_ p<0.05 vs VK_2_. _d_ p<0.05 vs ZA. _e_ p<0.05 vs VK_2_+ZA. _f_ p<0.05 vs VK_2_ to ZA. _g_ p<0.05 vs ZA to VK_2_.

**Table 2:** BMD, BMC and bone area values in the proximal metaphysic, distal metaphysic, bone diaphysis and the whole bone, and the blood lipids levels.

	Sham (n = 8)	OVX (n = 7)	VK_2_ (n = 8)	ZA (n = 11)	VK_2_+ZA (n = 9)	VK_2_ to ZA (n = 9)	ZA to VK_2_ (n = 8)
Proximal metaphysis							
BMC (mg)	80.44±7.72*	66.14±2.96	71.76±3.43	74.77±3.72*	67.32±6.53	80.29±6.32*	78.38±4.12*
Bone area (cm^2^)	0.44±0.02	0.43±0.02	0.43±0.02	0.44±0.02	0.43±0.04	0.45±0.03	0.47±0.02
BMD (mg/cm^2^)	184.10±17.95*	152.98±7.27	165.28±5.86*_f_	168.34±3.56*_f_	155.61±7.44	176.28±7.40*_cdg_	168.03±4.92*_f_
Distal metaphysis							
BMC (mg)	92.36±8.09*	72.32±6.41	83.59±4.80*	82.20±5.48	78.08±9.39	90.07±10.24*	84.02±7.66*
Bone area (cm^2^)	0.47±0.04	0.45±0.04	0.47±0.02	0.46±0.03	0.46±0.03	0.46±0.03	0.47±0.03
BMD (mg/cm^2^)	198.10±13.33*	160.17±8.31	176.69±8.39*_f_	179.83±7.54*_f_	168.76±13.51	194.92±15.10*_cdg_	179.01±9.50*_f_
Bone diaphysis							
BMC (mg)	178.12±12.63	151.64±22.46	164.75±19.26	166.49±16.89	164.24±22.87	160.92±21.40	159.85±22.01
Bone area (cm^2^)	1.18±0.05	1.11±0.14	1.18±0.11	1.16±0.11	1.16±0.13	1.10±0.14	1.12±0.20
BMD (mg/cm^2^)	150.66±7.55*	136.05±5.59	139.81±5.76	144.00±3.23	140.78±4.46	146.04±9.63*	143.76±10.10
Whole bone							
BMC (mg)	457.09±36.10*	345.70±47.63	406.35±54.38*	411.39±40.43*	365.83±30.74	422.88±28.85*	401.94±38.13
Bone area (cm^2^)	2.56±0.19	2.35±0.26	2.54±0.37	2.55±0.21	2.38±0.16	2.51±0.13	2.49±0.23
BMD (mg/cm^2^)	178.80±5.66*	150.51±5.44	160.31±5.43*_f_	160.95±3.41*_f_	153.81±7.76	168.23±3.63*_cdg_	161.24±4.36*_f_
Blood lipids analysis (mmol/L)							
TG	0.77±0.24	0.91±0.28	0.77±0.24	0.71±0.33	0.87±0.28	0.76±0.28	0.73±0.20
HDL-C	3.42±0.37	4.23±0.75	3.88±0.83	4.12±0.86	3.41±0.89	3.62±0.69	3.20±0.61
TC	0.84±0.13	1.14±0.15	1.04±0.23	1.11±0.27	0.94±0.22	1.02±0.11	0.97±0.12
LDL-C	0.07±0.01	0.11±0.04	0.09±0.02	0.08±0.03	0.09±0.03	0.10±0.03	0.10±0.03

Data are presented as the mean±SD. P<0.05 is considered statistically significant. A comparison of data between groups was performed using a one-way analysis of variance (ANOVA).Rank the data then using a one-way analysis of variance again, or nonparametric test was performed directly, when the variances were unequal. Dunnett’s two-tailed t-test is used when making multiple comparisons to the OVX group

* p<0.05 vs OVX. The SNK method is then used for multiple comparisons among the treatment groups that are found to be statistically significant in previous tests. _c_ p<0.05 vs VK_2_. _d_ p<0.05 vs ZA. _f_ p<0.05 vs VK_2_ to ZA. _g_ p<0.05 vs ZA to VK_2_.

**Fig 5:**
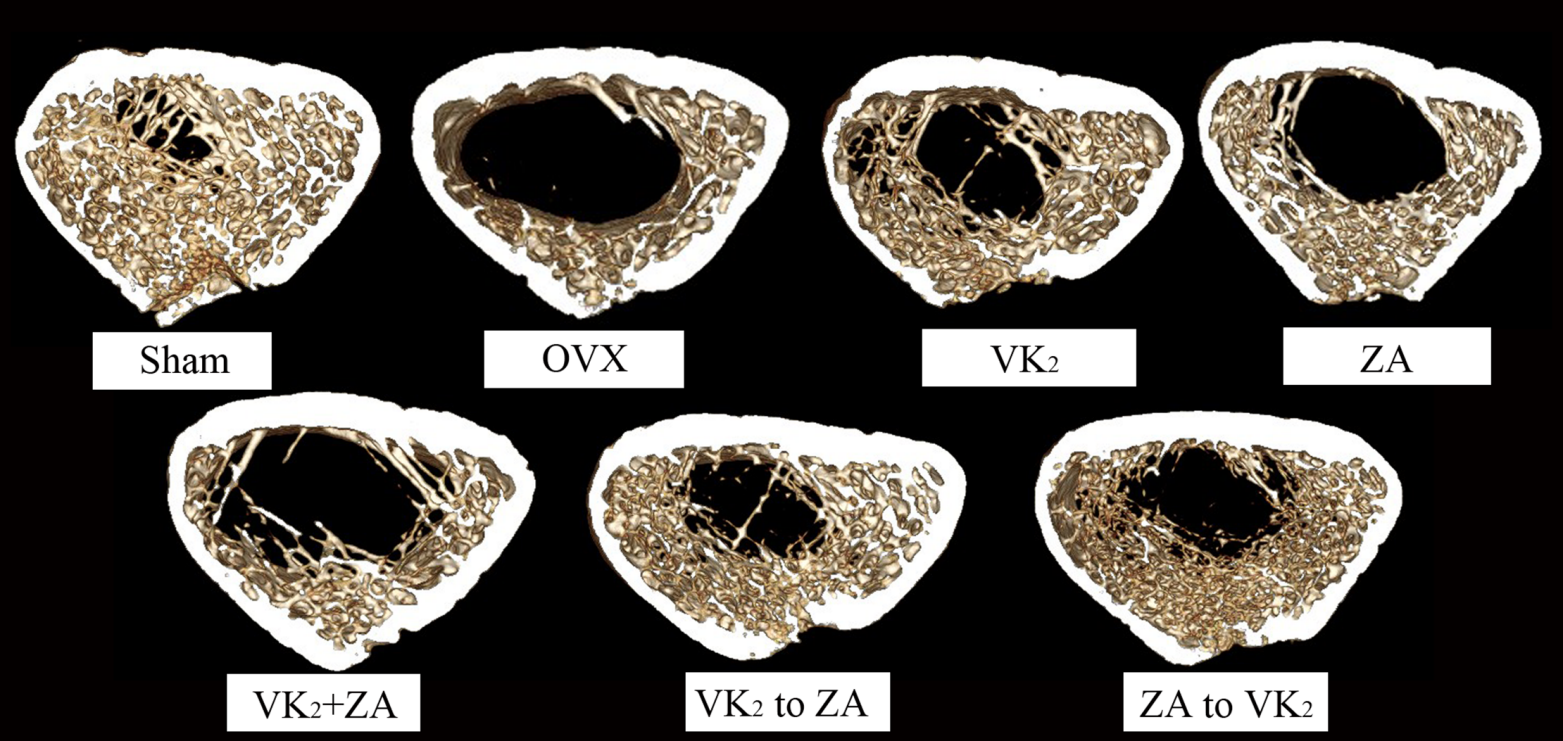
Effect of VK_2_ and/or ZA treatment on bone structure as revealed by micro-CT analysis of the trabecular bone structure in distal metaphysis. These pictures were representatives of the distal metaphysis between 100–250 layer proximal to the butterfly area after 12-week treatments.

**Fig 6:**
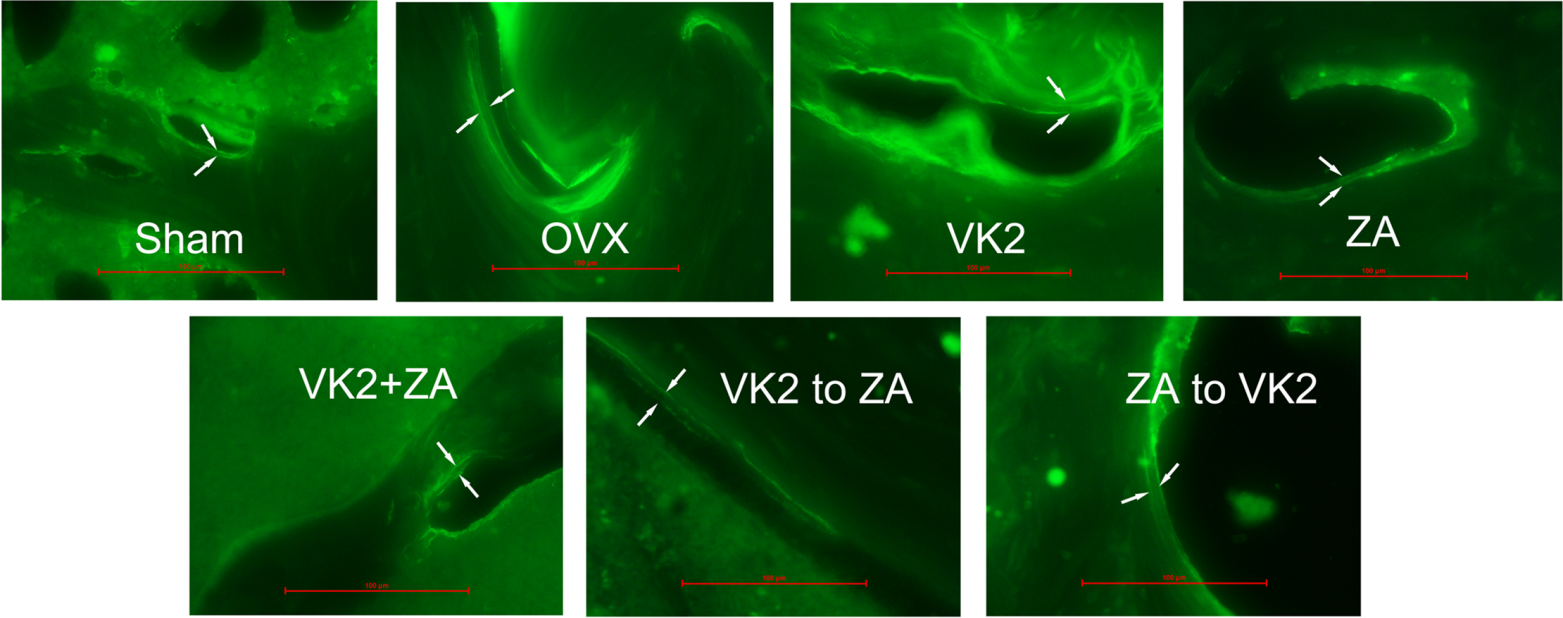
The surface-based bone turnover revealed by fluorescence microscope. These pictures were representatives of the double fluorescence labeling image.

Sequential protocol of VK_2_ to ZA also improved the trabecular bone structure and the anti-pressure strength of the bone compared with other protocols. In detail, a defeat feature in trabecular bone structure and bone biomechanical properties was shown in OVX animals (Table 1), and VK_2_, ZA, and ZA to VK_2_ protocols increased the BV/TV and Tb.N values, at the same decreased the BS/BV, Tb.Sp and Tb.Pf values. While the sequential protocol (VK_2_ to ZA) showed stronger effect than above three protocols, the VK_2_+ ZA protocol did not improve the bone condition at all compared with OVX model group. The bone structure as revealed by micro-CT (Fig 5) were in line with above mechanical or physical features. Beside the benefit revealed in trabecular bone structure, sequential protocol (VK_2_ to ZA) improved the elastic load, elastic stress, ultimate stress and Young’s modulus values, which of all contributed to improve the anti-pressure strength of the cortical bone (Table 1). Again, the indexes related to the osteoblasts function in vivo, including the percent labeled perimeter, mineral apposition rate and bone formation rate (Table 1, Fig 6) showed that the VK_2_ to ZA sequential protocol manifested its advantage over other protocols.

## Discussion

VK_2_ was well documented to promote bone formation either via direct anabolic effect on osteoblast or via anti-osteoclastic pathways [15, 16, 29], while ZA as an anticatabolic therapeutic for osteoporosis inhibited osteoblasts proliferation and mineralization. These frustrated outcomes were confirmed in our cellular/molecular studies. That is, ZA alone depressed osteoblasts proliferation in comparison with the VK_2_ alone at the comparable concentrations (i.e. 1/8, 1/4, 1/2, one, 2 or 4 folds of IC50). In addition, the osteoblast mineralization inhibition emerged when the ZA concentration was above 28.75μmol/L. So it was not absurd for us to try certain combinational protocols for these approved drugs to achieve a beneficial outcome. In current study, prior administration of VK_2_ equipped osteoblasts with an ability to antagonize the osteogenesis inhibition induced by ZA, and this beneficial effect was observed in both osteoblasts culture and OVX animal model. As with the mechanisms for the benefits of sequential protocol, a highly possible one was that VK_2_ may promote γ-carboxylation of osteocalcin, later of which directly affected bone metabolism in a lasting period even after the VK_2_ treatment was withdrawn. However, this hypothesis could not explain why the VK_2_+ZA protocol did not show beneficial effect over ZA treatment alone. The only guess was that, to equip osteoblasts with an ability to antagonize ZA-induced inhibition of osteoblasts proliferation and mineralization, VK_2_ has to be working when cells encountering ZA. Namely, once VK_2_ takes its protective effect, the formation of new bone will not be disturbed by ZA administration. The satisfactory outcomes manifested in the sequential protocol (VK_2_ to ZA) in vitro might be achieved by maintaining the protection capability induced by VK_2_, that is, up-regulating *Bcl-2* and *Cbfa1/Runx2* expression (moderately) while blocking *Bax* up-regulation. In addition, VK_2_ significantly blocked up-regulation of *Sost* expression thus contributed to enhancing the mineralization. Once the osteoblasts were equipped with this protection capability from VK_2_, they might become resistant to ZA’s inhibition. But, the anti-resorption effect of ZA would not be affected, thus leading to a net gain of bone mass.

As with the molecular mechanism underling the differential outcomes of above combinational protocols, we have to admit that our data was still preliminary and much more efforts are necessary, either to conform or defy above findings or proposal, and current study may just serve as an initiator. In brief, Runt-related transcription factor 2 (*Cbfa1/Runx2*) is a master transcription factor controlling osteoblasts differentiation. *Runx2*-null mice lack osteoblasts and fail to form functional bones [31]. However, over expression of Runx2 also induces low bone mass or even leads to spontaneous fracture [32]. In another aspect, sclerostin (*Sost*) was a Wnt signaling pathway inhibitor concerning bone metabolism [33] and down-regulation of the Sost expression helped to reactivate Wnt pathway hence promote bone formation [34]. Futhermore, Sost down-regulation also help to inhibit osteoclastogenesis by blocking the RANKL-OPG pathway and revert the bone loss [35]. Theoretically, since *Cbfa1/Runx2* binds the proximal *Sost* promoter and contributes to the *Sost* expression, the change of this pair of genes should be concerted. However, *Sost* expression might in turn decrease *Runx2* expression as a feedback [33]. This might be responsible for, at least in part, the observed discrepancy of these two genes in this study (Fig 4).

## Conclusion

VK_2_ pretreatment partially prevented the inhibition of ZA on bone formation parameters. The proposed mechanisms underlying such a beneficial protocol included anti-apoptosis and depression of *Sost* expression in osteoblasts by VK_2_, which partially counteracted ZA-reduced osteoblasts proliferation and mineralization inhibition, which in turn improved the osteoporosis therapeutic effects of ZA. The beneficial outcomes of VK_2_ pretreatment before ZA were confirmed in animal models. These findings supported that pretreatment with VK_2_ before ZA therapy might be recommended as a long term strategy for osteoporosis management. However, simultaneously protocol for VK_2_ and ZA (i.e. VK_2_+ZA) is not recommended due to lack of benefits over routine use of each single drug—at least at the doses of current study. Surely before actual performance of such transcription, extensive studies are deserved to investigate the pharmacodynamics of both agents, especially in tissues or bone surface. Also the applicability of current observation obtained with rats in human deserves more investigations.

## Supporting information

S1 File Supporting information. Figure A. Alkaline phosphatase staining method was utilized to identify osteoblasts. Osteoblasts were stained with alkaline phosphatase.
(DOCX)
