# Supplementary material for: Prior administration of vitamin K2 improves the therapeutic effects of zoledronic acid in ovariectomized rats by antagonizing zoledronic acid-induced inhibition of osteoblasts proliferation and mineralization
Source: PLoS One. 2018 Aug 20;13(8):e0202269. doi: 10.1371/journal.pone.0202269 (PMC6101397; doi:10.1371/journal.pone.0202269)
Supplement: S1 File — Figure A. Alkaline phosphatase staining method was utilized to identify osteoblasts. Osteoblasts were stained with alkaline phosphatase. (DOCX) [file pone.0202269.s001.docx]

**S1 File.**

**Supporting Information**

**Figure A. Alkaline phosphatase staining method was utilized to identify osteoblasts.**

To observe and identify the morphological feature of osteoblasts, alkaline phosphatase staining method (Azo-dye) was used. 2×10^4^ cells/well were seeded in complete growth medium in 24-well plate and cultured for a total of 144 hours. At the end of culture, cells were rinsed with PBS and fixed with 4% paraformaldehyde for 10 min, followed by staining with the solution (Beijing leagene biotech.co.,ltd, Beijing, China) according to the manufacturer at room temperature. Excess stain was carefully removed with distilled water. An inverted phase contrast microscope (Eclipse TS100, Nikon, Tokyo, Japan) was used to observe the morphology of and calcium deposition in cells. The images were captured and analyzed with NIS-Elements F (Nikon, Tokyo, Japan).


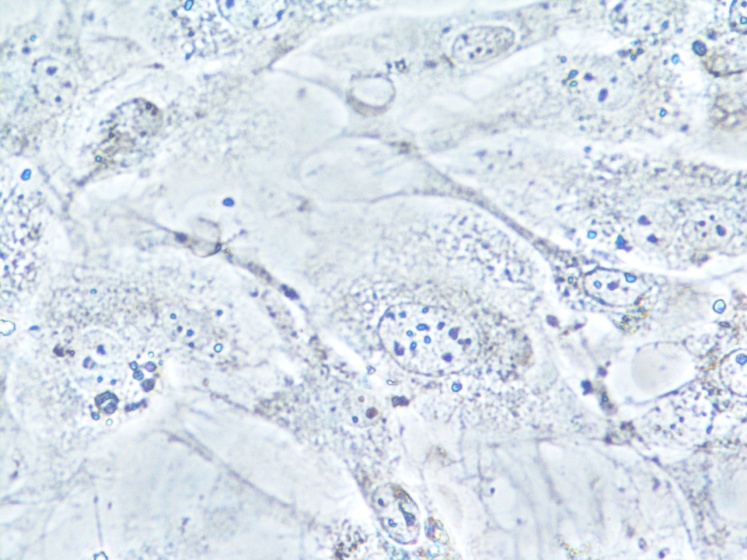


**Figure A**. Osteoblasts were stained with alkaline phosphatase.

**Table A. Details of statistical analysis and results (Figures in manuscript)**

**Table A. 1. Figure 3B in manuscript**

**VK_2_ 15 μmol/L, ZA 57.5 μmol/L**

|  | Control | VK_2_ | ZA | VK_2_+ZA | VK_2_ to ZA | ZA to VK_2_ |
| --- | --- | --- | --- | --- | --- | --- |
| VK_2_ | 0.000 |  | # | # | # | # |
| ZA | 0.000 | # |  |  | # |  |
| VK_2_+ZA | 0.000 | # |  |  | # |  |
| VK_2_ to ZA | 0.000 | # | # | # |  | # |
| ZA to VK_2_ | 0.000 | # |  |  | # |  |

Data are presented as the mean±SD. P<0.05 is considered statistically significant. A comparison of data between groups was performed using a one-way analysis of variance (ANOVA). Dunnett’s two-tailed t-test is used when making multiple comparisons to the control. The SNK method is then used for multiple comparisons among the treatment groups that are found to be statistically significant in previous tests. _#_ p<0.05.

**VK_2_ 7.5 μmol/L, ZA 28.75 μmol/L**

|  | Control | VK_2_ | ZA | VK_2_+ZA | VK_2_ to ZA | ZA to VK_2_ |
| --- | --- | --- | --- | --- | --- | --- |
| VK_2_ | 0.000 |  | # | # | # | # |
| ZA | 0.000 | # |  | # | # | # |
| VK_2_+ZA | 0.000 | # | # |  | # |  |
| VK_2_ to ZA | 0.037 | # | # | # |  | # |
| ZA to VK_2_ | 0.000 | # | # |  | # |  |

Data are presented as the mean±SD. P<0.05 is considered statistically significant. A comparison of data between groups was performed using a one-way analysis of variance (ANOVA). Dunnett’s two-tailed t-test is used when making multiple comparisons to the control. The SNK method is then used for multiple comparisons among the treatment groups that are found to be statistically significant in previous tests. _#_ p<0.05.

**Table A. 2. Figure 4 in manuscript.**

**Bcl-2**

|  | VK_2_ | ZA | VK_2_+ZA | VK_2_ to ZA | ZA to VK_2_ |
| --- | --- | --- | --- | --- | --- |
| VK_2_ |  | 0.001 |  |  |  |
| ZA |  |  |  |  |  |
| VK_2_+ZA |  | 0.134 |  |  |  |
| VK_2_ to ZA |  | 0.095 |  |  |  |
| ZA to VK_2_ |  | 0.112 |  |  |  |

Data are presented as the mean±SD. P<0.05 is considered statistically significant. A comparison of data between groups was performed using a one-way analysis of variance (ANOVA). Dunnett’s two-tailed t-test is used when making multiple comparisons to the ZA. The SNK method is then used for multiple comparisons among the treatment groups that are found to be statistically significant in previous tests. _#_ p<0.05.

**Bax**

|  | VK_2_ | ZA | VK_2_+ZA | VK_2_ to ZA | ZA to VK_2_ |
| --- | --- | --- | --- | --- | --- |
| VK_2_ |  | 0.000 |  | # | # |
| ZA |  |  |  |  |  |
| VK_2_+ZA |  | 0.120 |  |  |  |
| VK_2_ to ZA | # | 0.000 |  |  | # |
| ZA to VK_2_ | # | 0.024 |  | # |  |

Data are presented as the mean±SD. P<0.05 is considered statistically significant. A comparison of data between groups was performed using a one-way analysis of variance (ANOVA). Dunnett’s two-tailed t-test is used when making multiple comparisons to the ZA. The SNK method is then used for multiple comparisons among the treatment groups that are found to be statistically significant in previous tests. _#_ p<0.05.

**Bcl-2/Bax**

|  | VK_2_ | ZA | VK_2_+ZA | VK_2_ to ZA | ZA to VK_2_ |
| --- | --- | --- | --- | --- | --- |
| VK_2_ |  | 0.000 |  | # | # |
| ZA |  |  |  |  |  |
| VK_2_+ZA |  | 0.120 |  |  | # |
| VK_2_ to ZA | # | 0.000 |  |  |  |
| ZA to VK_2_ | # | 0.001 |  | # |  |

Data are presented as the mean±SD. P<0.05 is considered statistically significant. A comparison of data between groups was performed using a one-way analysis of variance (ANOVA). Dunnett’s two-tailed t-test is used when making multiple comparisons to the ZA. The SNK method is then used for multiple comparisons among the treatment groups that are found to be statistically significant in previous tests. _#_ p<0.05.

**SOST**

|  | VK_2_ | ZA | VK_2_+ZA | VK_2_ to ZA | ZA to VK_2_ |
| --- | --- | --- | --- | --- | --- |
| VK_2_ |  | 0.017 | # |  |  |
| ZA |  |  |  |  |  |
| VK_2_+ZA | # | 0.000 |  | # |  |
| VK_2_ to ZA |  | 0.026 | # |  |  |
| ZA to VK_2_ |  | 0.705 |  |  |  |

Data are presented as the mean±SD. P<0.05 is considered statistically significant. A comparison of data between groups was performed using a one-way analysis of variance (ANOVA). Dunnett’s two-tailed t-test is used when making multiple comparisons to the ZA. The SNK method is then used for multiple comparisons among the treatment groups that are found to be statistically significant in previous tests. _#_ p<0.05.

**RUNX2**

|  | VK_2_ | ZA | VK_2_+ZA | VK_2_ to ZA | ZA to VK_2_ |
| --- | --- | --- | --- | --- | --- |
| VK_2_ |  | 0.000 | # | # | # |
| ZA |  |  |  |  |  |
| VK_2_+ZA | # | 0.000 |  | # | # |
| VK_2_ to ZA | # | 0.011 | # |  | # |
| ZA to VK_2_ | # | 0.000 | # | # |  |

Data are presented as the mean±SD. P<0.05 is considered statistically significant. A comparison of data between groups was performed using a one-way analysis of variance (ANOVA). Dunnett’s two-tailed t-test is used when making multiple comparisons to the ZA. The SNK method is then used for multiple comparisons among the treatment groups that are found to be statistically significant in previous tests. _#_ p<0.05

**Table B. Details of statistical analysis and results (Table 1 in manuscript)**

**Table B. 1. Surface-based bone turnover data (Table 1 in manuscript)**

**%L.Pm(%)**

|  | sham | OVX | VK_2_ | ZA | VK_2_+ZA | VK_2_ to ZA | ZA to VK_2_ |
| --- | --- | --- | --- | --- | --- | --- | --- |
| sham |  | 0.000 |  |  |  |  |  |
| OVX | 0.000 |  | 0.000 | 0.042 | 0.000 | 0.010 | 1.000 |
| VK_2_ |  | 0.000 |  | # | # | # |  |
| ZA |  | 0.042 | # |  | # | # |  |
| ZA+VK_2_ |  | 0.000 | # | # |  | # |  |
| VK_2_ to ZA |  | 0.010 | # | # | # |  |  |
| ZA to VK_2_ |  | 1.000 |  |  |  |  |  |

Data are presented as the mean±SD. P<0.05 is considered statistically significant. A comparison of data between groups was performed using a one-way analysis of variance (ANOVA-RANK). Dunnett’s two-tailed t-test is used when making multiple comparisons to the OVX. The SNK method is then used for multiple comparisons among the treatment groups that are found to be statistically significant in previous tests. _#_ p<0.05.

**MAR(μm/d)**

|  | sham | OVX | VK_2_ | ZA | VK_2_+ZA | VK_2_ to ZA | ZA to VK_2_ |
| --- | --- | --- | --- | --- | --- | --- | --- |
| sham |  | 0.000 |  |  |  |  |  |
| OVX | 0.000 |  | 0.000 | 0.143 | 0.000 | 0.043 | 0.996 |
| VK_2_ |  | 0.000 |  |  | # | # |  |
| ZA |  | 0.143 |  |  |  |  |  |
| ZA+VK_2_ |  | 0.000 | # |  |  | # |  |
| VK_2_ to ZA |  | 0.043 | # |  | # |  |  |
| ZA to VK_2_ |  | 0.996 |  |  |  |  |  |

Data are presented as the mean±SD. P<0.05 is considered statistically significant. A comparison of data between groups was performed using a one-way analysis of variance (ANOVA). Dunnett’s two-tailed t-test is used when making multiple comparisons to the OVX. The SNK method is then used for multiple comparisons among the treatment groups that are found to be statistically significant in previous tests. _#_ p<0.05.

**BFR(μm/d*%)**

|  | sham | OVX | VK_2_ | ZA | VK_2_+ZA | VK_2_ to ZA | ZA to VK_2_ |
| --- | --- | --- | --- | --- | --- | --- | --- |
| sham |  | 0.000 |  |  |  |  |  |
| OVX | 0.000 |  | 0.000 | 0.041 | 0.000 | 0.005 | 0.996 |
| VK_2_ |  | 0.000 |  | # | # | # |  |
| ZA |  | 0.041 | # |  | # | # |  |
| ZA+VK_2_ |  | 0.000 | # | # |  | # |  |
| VK_2_ to ZA |  | 0.005 | # | # | # |  |  |
| ZA to VK_2_ |  | 0.996 |  |  |  |  |  |

Data are presented as the mean±SD. P<0.05 is considered statistically significant. A comparison of data between groups was performed using a one-way analysis of variance (ANOVA-RANK). Dunnett’s two-tailed t-test is used when making multiple comparisons to the OVX. The SNK method is then used for multiple comparisons among the treatment groups that are found to be statistically significant in previous tests. _#_ p<0.05.

**Table B. 2. Bone calcium content (Table 1 in manuscript)**

**Bone calcium content (mg/g)**

|  | sham | OVX | VK_2_ | ZA | VK_2_+ZA | VK_2_ to ZA | ZA to VK_2_ |
| --- | --- | --- | --- | --- | --- | --- | --- |
| sham |  | 0.000 |  |  |  |  |  |
| OVX | 0.000 |  | 0.021 | 0.006 | 0.181 | 0.000 | 0.059 |
| VK_2_ |  | 0.021 |  |  |  | # |  |
| ZA |  | 0.006 |  |  |  | # |  |
| ZA+VK_2_ |  | 0.181 |  |  |  |  |  |
| VK_2_ to ZA |  | 0.000 | # | # |  |  |  |
| ZA to VK_2_ |  | 0.059 |  |  |  |  |  |

Data are presented as the mean±SD. P<0.05 is considered statistically significant. A comparison of data between groups was performed using a one-way analysis of variance (ANOVA). Dunnett’s two-tailed t-test is used when making multiple comparisons to the OVX. The SNK method is then used for multiple comparisons among the treatment groups that are found to be statistically significant in previous tests. _#_ p<0.05.

**Table B. 3. The trabecular bone parameters of the distal metaphysic (Table 1 in manuscript)**

**BV/TV (%)**

|  | sham | OVX | VK_2_ | ZA | VK_2_+ZA | VK_2_ to ZA | ZA to VK_2_ |
| --- | --- | --- | --- | --- | --- | --- | --- |
| sham |  | 0.000 |  |  |  |  |  |
| OVX | 0.000 |  | 0.004 | 0.000 | 0.919 | 0.000 | 0.018 |
| VK_2_ |  | 0.004 |  |  |  | # |  |
| ZA |  | 0.000 |  |  |  | # |  |
| ZA+VK_2_ |  | 0.919 |  |  |  |  |  |
| VK_2_ to ZA |  | 0.000 | # | # |  |  | # |
| ZA to VK_2_ |  | 0.018 |  |  |  | # |  |

Data are presented as the mean±SD. P<0.05 is considered statistically significant. A comparison of data between groups was performed using a one-way analysis of variance (ANOVA). Dunnett’s two-tailed t-test is used when making multiple comparisons to the OVX. The SNK method is then used for multiple comparisons among the treatment groups that are found to be statistically significant in previous tests. _#_ p<0.05.

**BS/BV (1/mm)**

|  | sham | OVX | VK_2_ | ZA | VK_2_+ZA | VK_2_ to ZA | ZA to VK_2_ |
| --- | --- | --- | --- | --- | --- | --- | --- |
| sham |  | 0.013 |  |  |  |  |  |
| OVX | 0.013 |  | 0.439 | 0.203 | 0.971 | 0.049 | 0.674 |
| VK_2_ |  | 0.439 |  |  |  |  |  |
| ZA |  | 0.203 |  |  |  |  |  |
| ZA+VK_2_ |  | 0.971 |  |  |  |  |  |
| VK_2_ to ZA |  | 0.049 |  |  |  |  |  |
| ZA to VK_2_ |  | 0.674 |  |  |  |  |  |

Data are presented as the mean±SD. P<0.05 is considered statistically significant. A comparison of data between groups was performed using a one-way analysis of variance (ANOVA). Dunnett’s two-tailed t-test is used when making multiple comparisons to the OVX.

**Tb.N (1/mm)**

|  | sham | OVX | VK_2_ | ZA | VK_2_+ZA | VK_2_ to ZA | ZA to VK_2_ |
| --- | --- | --- | --- | --- | --- | --- | --- |
| sham |  | 0.000 |  |  |  |  |  |
| OVX | 0.000 |  | 0.002 | 0.000 | 0.753 | 0.000 | 0.000 |
| VK_2_ |  | 0.002 |  |  |  | # |  |
| ZA |  | 0.000 |  |  |  | # |  |
| ZA+VK_2_ |  | 0.753 |  |  |  |  |  |
| VK_2_ to ZA |  | 0.000 | # | # |  |  | # |
| ZA to VK_2_ |  | 0.000 |  |  |  | # |  |

Data are presented as the mean±SD. P<0.05 is considered statistically significant. A comparison of data between groups was performed using a one-way analysis of variance (ANOVA). Dunnett’s two-tailed t-test is used when making multiple comparisons to the OVX. The SNK method is then used for multiple comparisons among the treatment groups that are found to be statistically significant in previous tests. _#_ p<0.05.

**Tb.Sp (µm)**

|  | sham | OVX | VK_2_ | ZA | VK_2_+ZA | VK_2_ to ZA | ZA to VK_2_ |
| --- | --- | --- | --- | --- | --- | --- | --- |
| sham |  | 0.000 | 0.003 | 0.005 | 0.000 | 0.477 | 0.005 |
| OVX | 0.000 |  | 0.019 | 0.004 | 0.690 | 0.000 | 0.014 |
| VK_2_ | 0.003 | 0.019 |  | 0.727 | 0.044 | 0.025# | 0.906 |
| ZA | 0.005 | 0.004 | 0.727 |  | 0.012 | 0.040# | 0.825 |
| ZA+VK_2_ | 0.000 | 0.690 | 0.044 | 0.012 |  | 0.000 | 0.033 |
| VK_2_ to ZA | 0.477 | 0.000 | 0.025# | 0.040# | 0.000 |  | 0.034# |
| ZA to VK_2_ | 0.005 | 0.014 | 0.906 | 0.825 | 0.033 | 0.034# |  |

Data are presented as the mean±SD. P<0.05 is considered statistically significant. A comparison of data between groups was performed using a Kruskal-Wallis test (Non-parametric test). Dunnett’s two-tailed t-test is used when making multiple comparisons to the OVX. The SNK method is then used for multiple comparisons among the treatment groups that are found to be statistically significant in previous tests. _#_ p<0.05.

**Tb.Pf (1/mm)**

|  | sham | OVX | VK_2_ | ZA | VK_2_+ZA | VK_2_ to ZA | ZA to VK_2_ |
| --- | --- | --- | --- | --- | --- | --- | --- |
| sham |  | 0.000 | 0.001 | 0.001 | 0.000 | 0.300 | 0.001 |
| OVX | 0.000 |  | 0.011 | 0.002 | 0.116 | 0.000 | 0.010 |
| VK_2_ | 0.001 | 0.011 |  | 0.714 | 0.321 | 0.020# | 0.976 |
| ZA | 0.001 | 0.002 | 0.714 |  | 0.152 | 0.033# | 0.738 |
| ZA+VK_2_ | 0.000 | 0.116 | 0.321 | 0.152 |  | 0.001 | 0.307 |
| VK_2_ to ZA | 0.300 | 0.000 | 0.020# | 0.033# | 0.001 |  | 0.022# |
| ZA to VK_2_ | 0.001 | 0.010 | 0.976 | 0.738 | 0.307 | 0.022# |  |

Data are presented as the mean±SD. P<0.05 is considered statistically significant. A comparison of data between groups was performed using a Kruskal-Wallis test (Non-parametric test). Dunnett’s two-tailed t-test is used when making multiple comparisons to the OVX. The SNK method is then used for multiple comparisons among the treatment groups that are found to be statistically significant in previous tests. _#_ p<0.05.

**Table C. Details of statistical analysis and results (Table 2 in manuscript)**

**Table C. 1. Proximal metaphysis (Table 2 in manuscript)**

**BMC**

|  | sham | OVX | VK_2_ | ZA | VK_2_+ZA | VK_2_ to ZA | ZA to VK_2_ |
| --- | --- | --- | --- | --- | --- | --- | --- |
| sham |  | 0.000 |  |  |  |  |  |
| OVX | 0.000 |  | 0.173 | 0.008 | 0.994 | 0.000 | 0.000 |
| VK_2_ |  | 0.173 |  |  |  |  |  |
| ZA |  | 0.008 |  |  |  |  |  |
| ZA+VK_2_ |  | 0.994 |  |  |  |  |  |
| VK_2_ to ZA |  | 0.000 |  |  |  |  |  |
| ZA to VK_2_ |  | 0.000 |  |  |  |  |  |

Data are presented as the mean±SD. P<0.05 is considered statistically significant. A comparison of data between groups was performed using a one-way analysis of variance (ANOVA). Dunnett’s two-tailed t-test is used when making multiple comparisons to the OVX. The SNK method is then used for multiple comparisons among the treatment groups that are found to be statistically significant in previous tests. _#_ p<0.05.

**BMD**

|  | sham | OVX | VK_2_ | ZA | VK_2_+ZA | VK_2_ to ZA | ZA to VK_2_ |
| --- | --- | --- | --- | --- | --- | --- | --- |
| sham |  | 0.000 |  |  |  |  |  |
| OVX | 0.000 |  | 0.004 | 0.000 | 0.971 | 0.000 | 0.001 |
| VK_2_ |  | 0.004 |  |  |  | # |  |
| ZA |  | 0.000 |  |  |  | # |  |
| ZA+VK_2_ |  | 0.971 |  |  |  |  |  |
| VK_2_ to ZA |  | 0.000 | # | # |  |  | # |
| ZA to VK_2_ |  | 0.001 |  |  |  | # |  |

Data are presented as the mean±SD. P<0.05 is considered statistically significant. A comparison of data between groups was performed using a one-way analysis of variance (ANOVA). Dunnett’s two-tailed t-test is used when making multiple comparisons to the OVX. The SNK method is then used for multiple comparisons among the treatment groups that are found to be statistically significant in previous tests. _#_ p<0.05.

**Table C. 2. Distal metaphysis (Table 2 in manuscript)**

**BMC**

|  | sham | OVX | VK_2_ | ZA | VK_2_+ZA | VK_2_ to ZA | ZA to VK_2_ |
| --- | --- | --- | --- | --- | --- | --- | --- |
| sham |  | 0.000 |  |  |  |  |  |
| OVX | 0.000 |  | 0.039 | 0.062 | 0.498 | 0.000 | 0.030 |
| VK_2_ |  | 0.039 |  |  |  |  |  |
| ZA |  | 0.062 |  |  |  |  |  |
| ZA+VK_2_ |  | 0.498 |  |  |  |  |  |
| VK_2_ to ZA |  | 0.000 |  |  |  |  |  |
| ZA to VK_2_ |  | 0.030 |  |  |  |  |  |

Data are presented as the mean±SD. P<0.05 is considered statistically significant. A comparison of data between groups was performed using a one-way analysis of variance (ANOVA). Dunnett’s two-tailed t-test is used when making multiple comparisons to the OVX. The SNK method is then used for multiple comparisons among the treatment groups that are found to be statistically significant in previous tests. _#_ p<0.05.

**BMD**

|  | sham | OVX | VK_2_ | ZA | VK_2_+ZA | VK_2_ to ZA | ZA to VK_2_ |
| --- | --- | --- | --- | --- | --- | --- | --- |
| sham |  | 0.000 |  |  |  |  |  |
| OVX | 0.000 |  | 0.022 | 0.005 | 0.472 | 0.000 | 0.014 |
| VK_2_ |  | 0.022 |  |  |  | # |  |
| ZA |  | 0.005 |  |  |  | # |  |
| ZA+VK_2_ |  | 0.472 |  |  |  |  |  |
| VK_2_ to ZA |  | 0.000 | # | # |  |  | # |
| ZA to VK_2_ |  | 0.014 |  |  |  | # |  |

Data are presented as the mean±SD. P<0.05 is considered statistically significant. A comparison of data between groups was performed using a one-way analysis of variance (ANOVA). Dunnett’s two-tailed t-test is used when making multiple comparisons to the OVX. The SNK method is then used for multiple comparisons among the treatment groups that are found to be statistically significant in previous tests. _#_ p<0.05.

**Table C. 3. Bone diaphysis (Table 2 in manuscript)**

**BMD**

|  | sham | OVX | VK_2_ | ZA | VK_2_+ZA | VK_2_ to ZA | ZA to VK_2_ |
| --- | --- | --- | --- | --- | --- | --- | --- |
| sham |  | 0.001 |  |  |  |  |  |
| OVX | 0.001 |  | 0.801 | 0.105 | 0.589 | 0.030 | 0.173 |
| VK_2_ |  | 0.801 |  |  |  |  |  |
| ZA |  | 0.105 |  |  |  |  |  |
| ZA+VK_2_ |  | 0.589 |  |  |  |  |  |
| VK_2_ to ZA |  | 0.030 |  |  |  |  |  |
| ZA to VK_2_ |  | 0.173 |  |  |  |  |  |

Data are presented as the mean±SD. P<0.05 is considered statistically significant. A comparison of data between groups was performed using a one-way analysis of variance (ANOVA). Dunnett’s two-tailed t-test is used when making multiple comparisons to the OVX. The SNK method is then used for multiple comparisons among the treatment groups that are found to be statistically significant in previous tests. _#_ p<0.05.

**Table C. 4. Whole bone (Table 2 in manuscript)**

**BMC**

|  | sham | OVX | VK_2_ | ZA | VK_2_+ZA | VK_2_ to ZA | ZA to VK_2_ |
| --- | --- | --- | --- | --- | --- | --- | --- |
| sham |  | 0.000 |  |  |  |  |  |
| OVX | 0.000 |  | 0.028 | 0.008 | 0.837 | 0.005 | 0.058 |
| VK_2_ |  | 0.028 |  |  |  |  |  |
| ZA |  | 0.008 |  |  |  |  |  |
| ZA+VK_2_ |  | 0.837 |  |  |  |  |  |
| VK_2_ to ZA |  | 0.005 |  |  |  |  |  |
| ZA to VK_2_ |  | 0.058 |  |  |  |  |  |

Data are presented as the mean±SD. P<0.05 is considered statistically significant. A comparison of data between groups was performed using a one-way analysis of variance (ANOVA). Dunnett’s two-tailed t-test is used when making multiple comparisons to the OVX. The SNK method is then used for multiple comparisons among the treatment groups that are found to be statistically significant in previous tests. _#_ p<0.05.

**BMD**

|  | sham | OVX | VK_2_ | ZA | VK_2_+ZA | VK_2_ to ZA | ZA to VK_2_ |
| --- | --- | --- | --- | --- | --- | --- | --- |
| sham |  | 0.000 |  |  |  |  |  |
| OVX | 0.000 |  | 0.004 | 0.001 | 0.662 | 0.000 | 0.001 |
| VK_2_ |  | 0.004 |  |  |  | # |  |
| ZA |  | 0.001 |  |  |  | # |  |
| ZA+VK_2_ |  | 0.662 |  |  |  |  |  |
| VK_2_ to ZA |  | 0.000 | # | # |  |  | # |
| ZA to VK_2_ |  | 0.002 |  |  |  | # |  |

Data are presented as the mean±SD. P<0.05 is considered statistically significant. A comparison of data between groups was performed using a one-way analysis of variance (ANOVA). Dunnett’s two-tailed t-test is used when making multiple comparisons to the OVX. The SNK method is then used for multiple comparisons among the treatment groups that are found to be statistically significant in previous tests. _#_ p<0.05.

**Table D. Abbreviations and annotations**

| **Abbreviation** | **Annotation** |
| --- | --- |
| TG | Triglyceride |
| HDL-C | High density lipoprotein cholesterol |
| TC | Total cholesterol |
| LDL-C | Low density lipoprotein cholesterol |
| BV/TV(%) | Bone volume fraction (%) |
| BS/BV(1/mm) | Bone specific surface (1/mm) |
| Tb.Th (µm) | Trabecular thickness (µm) |
| Tb.N (1/mm) | Trabecular number (1/mm) |
| Tb.Sp (µm) | Trabecular separation (µm) |
| Tb.Pf (1/mm) | Trabecular bone pattern factor (1/mm) |
| CSMI (mm^4^) | The cross-sectional moment of inertia (mm^4^) |
| BMC(mg) | Bone mineral content (mg) |
| BMD(mg/cm^2^) | Bone mineral density (mg/cm^2^) |
| VK2 | Vitamin K2 |
| ZA | Zoledronic acid |
| IC50 | 50% inhibition |
| CI | Combination index |
| Bcl-2 | B-cell lymphoma-2 |
| Bax | Bcl-2 Assacitated X protein |
| Runx2 | Runt related transcription factor 2 |
| Sost | Sclerostin |
